# Dataset on performance of large-scale vanadium redox flow batteries with serpentine flow fields

**DOI:** 10.1016/j.dib.2021.106835

**Published:** 2021-02-04

**Authors:** Ravendra Gundlapalli, Sreenivas Jayanti

**Affiliations:** Department of Chemical Engineering and DST-Solar Energy Harnessing Center, IIT Madras, Chennai 600036, India

**Keywords:** Vanadium redox flow battery, Flow fields, Electrode compression, Electrolyte distribution, Concentration over-potential, Life cycle behavior

## Abstract

The dataset presented in this article are related to research articles “Effect of electrolyte convection velocity in the electrode on the performance of vanadium redox flow battery cells with serpentine flow fields” [1] and “Effect of channel dimensions of serpentine flow fields on the performance of a vanadium redox flow battery” [2]. The combined dataset on the pressure drop and electrochemical behavior of the vanadium flow battery cells with active areas of 400 cm^2^, 900 cm^2^ and 1500 cm^2^ were obtained using battery life cycler for the circulation of vanadium electrolyte of concentration 1.61 M VOSO_4_ dissolved in 5 M H_2_SO_4_. The cells were designed with various combinations of flow-channel dimensions of serpentine flow field and the electrochemical performance has been obtained at various flow rates and current densities. In addition to the experimental data, computational fluid dynamics simulations have been performed to investigate the electrolyte distribution across the cell. The shared data enables the reader of research articles to delve into the life cycle behavior at various operating conditions and emphasize the importance of flow-channel dimensions, flow rate and uniform distribution of electrolyte in combating the concentration over-potential.

## Specifications Table

**Table:** 

Subject	Energy
Specific subject area	Redox flow batteries for application in medium to large scale energy storage for both off-grid and on-grid operations
Type of data	Image, Graph, Figure, Table and excel file
How data were acquired	Electrochemical data: Battery life cycler of the make Bitrode: LCN2–100–48 having inbuilt Visual LCN Software, biological: BCS-815 with inbuilt software BT-Lab Pressure drop: U-tube manometer
Data format	Raw, analyzed (processed)
Parameters for data collection	The electrolyte temperature was maintained constant at room temperature (26 °C) and the nitrogen gas was purged continuously through negative solution. The electrochemical data of life cycles were collected under constant current operation for both the charge and discharge conditions.
Description of data collection	By using the parameters specified in the above row, the data were collected with pre-set voltage cut-off 1.8 V and 0.8 V for charge and discharge conditions respectively.
Data source location	Institution: Indian Institute of Technology Madras City/Town/Region: Chennai, Country: India
Data accessibility	The raw and processed data is available as repository on the Mendeley Data and can be accessed using the following URL http://dx.doi.org/10.17632/vp4bwt7d2c.2
Related research article	[1] Ravendra Gundlapalli, Sreenivas Jayanti, Effect of Electrolyte Convection Velocity in the Electrode on the Performance of Vanadium Redox Flow Battery Cells with Serpentine Flow Field, Journal of Energy Storage, 30 (2020), 101,516, https://doi.org/10.1016/j.est.2020.101516 [2] Ravendra Gundlapalli, Sreenivas Jayanti, Effect of channel dimensions of serpentine flow fields on the performance of a vanadium redox flow battery, Journal of Energy Storage, 23 (2019) 148–158, https://doi.org/10.1016/j.est.2019.03.014

## Value of the Data

• The data presents charge-discharge life cycle behavior of the vanadium redox flow battery along with pressure drop measurements at various flow rates and current densities for several combinations of channel dimensions of serpentine flow field on a cell area of 400 cm^2^ and systematic scale-up studies over the increased cell areas 416, 918 and 1495 cm^2^.
• The data also presents velocity profiles across the porous electrode and flow-channels which are predicted by computational fluid dynamics (CFD) simulations.
• Any researcher, engineer or industry interested in developing large-scale flow battery would benefit from the data presented in this article.
• The data are useful to evaluate the polarization behavior of the cell as a function of state of charge of the electrolyte for a given flow rate and current density.
• The data can be used to develop experimental protocols and to improve the geometry of flow field to achieve the optimal electrochemical performance ensuing good electrolyte distribution with low parasitic pump losses.

## 1 Data Description

Contents include one Table, seven Figures and three Excel sheets. The raw data recorded in BT-Lab software (biological) and Visual LCN software (Bitrode) is exported to excel format. Each operating condition consists three life-cycles and the average of them is presented in the excel sheet from which the other parameters like capacity, energy density and efficiency are calculated. The excel sheets have original raw data exported from the data recording software and also have averaged life cycles with processed data for the electrochemical parameters.

Table 1: Contains the summary of design of cells with serpentine flow-channel combinations and scale-up studies for which the data is presented in this data article.

**Table 1:** Summary of the VRFB cells studied with serpentine flow field.

			Experimental conditions		

Active area (cm^2^)	Flow-channel dimensions (cw, rw, cd), mm	Electrode compression ratio (%)	Flow rate (ml/min.cm^2^)	Current density (mA/cm^2^)	Research article
400	(3,2,3); (3,3,3); (4,2,3); (4,3,3); (5,2,3); (5,3,3); (4,2,2); (3,3,4)	50	0.36	60	2
	(5,2,3)	35	0.18 to 0.84	45 to 120	1
900	(4,3,3), (5,2,3)	50	0.12 to 0.65	60	2
	(5,2,3)	35	0.18 to 0.84	45 to 120	1
1495	(5,2,3)	35	0.18 to 0.84	45 to 120	1

**Note**: cw - channel width, rw - rib width, cd - channel depth.

**Fig. 1:**
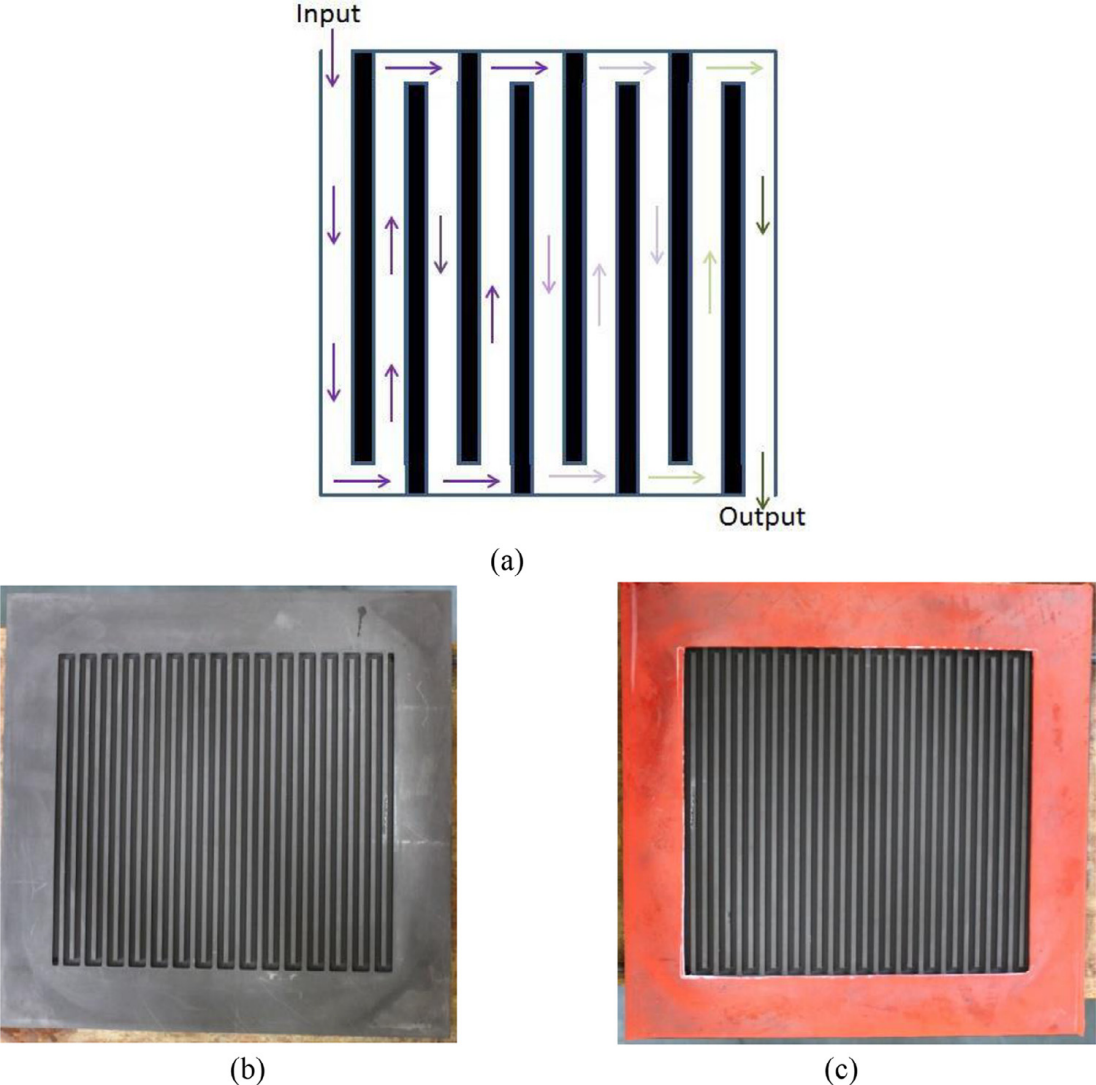
(a) Schematic of serpentine flow field, (b) flow field engraved on graphite plate and (c) silicone gasket overlaid on the overhead area of graphite plate.

Fig. 1: Shows the schematic of serpentine flow field along the image of grooved flow field and overlaid gasket on the active and overhead areas of graphite plate, respectively.

**Fig. 2:**
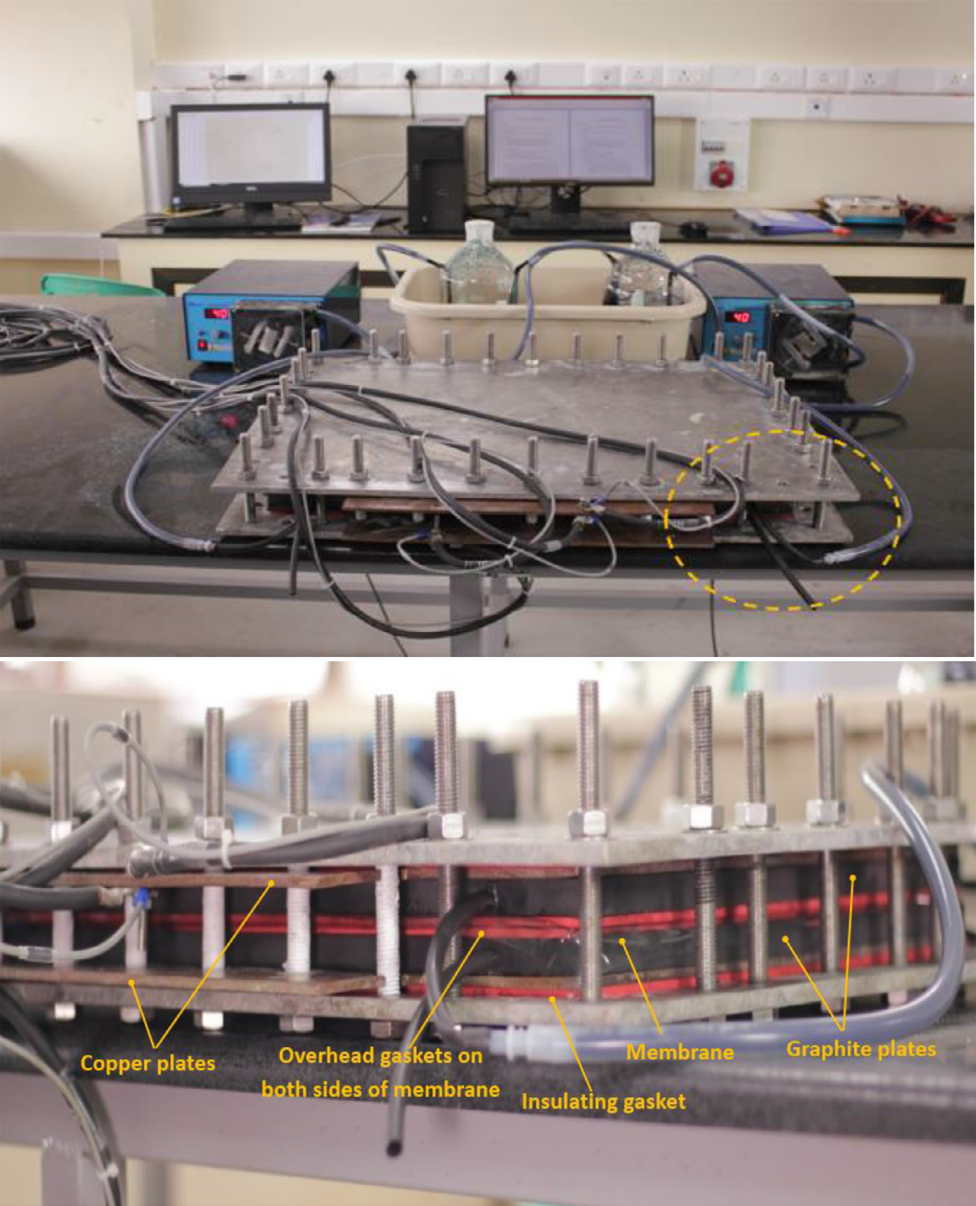
Image of assembled single vanadium redox flow battery cell in operation (top) and zoomed version of the dashed-circle (bottom).

Fig. 2: Shows the image of an assembled cell in operation with components identified.

**Fig. 3:**
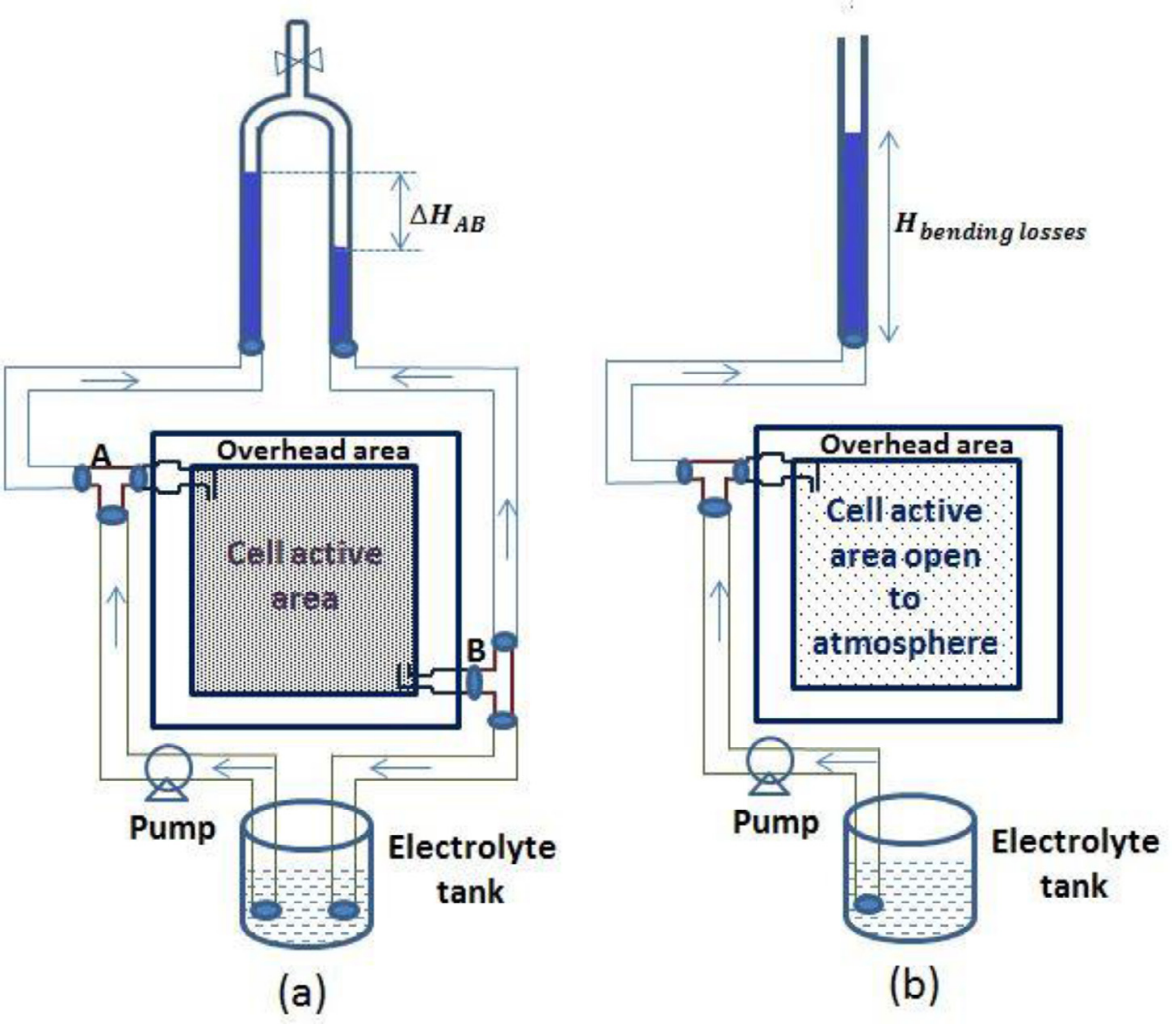
Schematic representation of measurement of (a) pressure drop in single cell VRFB with inverted U-tube manometer and (b) bending losses at inlet/outlet of cell with piezometer.

Fig. 3: Shows the schematic of measurement of total pressure drop in cell using manometer and bending losses in inlet-outlet sections of the cell using piezometer.

**Fig. 4:**
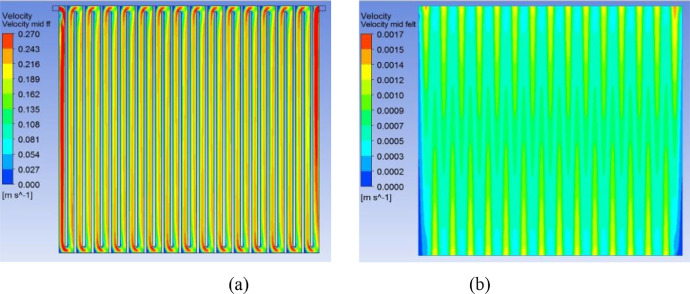
Predicted velocity contours for the cell area 416 cm^2^ for an inlet flow rate 0.30 ml/min.cm^2^ along a plane created at mid-depth of (a) flow-channel region (b) porous felt region.

**Fig. 5:**
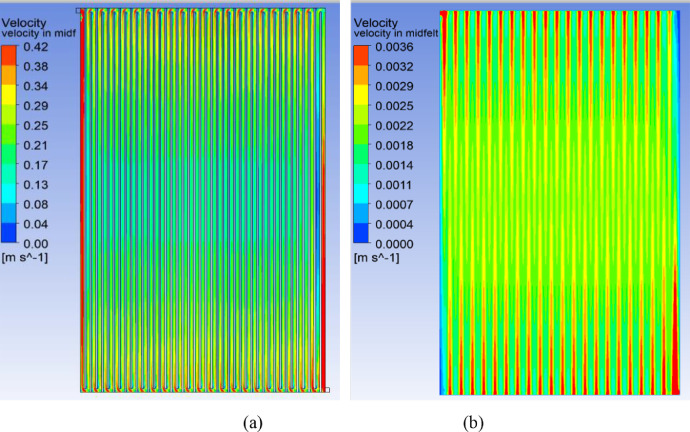
Predicted velocity contours for the cell area 1495 cm^2^ for an inlet flow rate 0.30 ml/min.cm^2^ along a plane created at mid-depth of (a) flow-channel and (b) porous felt regions.

Figs. 4 and 5: CFD predicted velocity contours for cell areas of 416 cm^2^ and 1495 cm^2^ for an inlet flow rate 0.30 ml/min.cm^2^ along a plane created at mid-depth of flow-channel region and porous felt region. Similar contours for the cell of active area 918 cm^2^ have already been published in the research article [1].

**Fig. 6:**
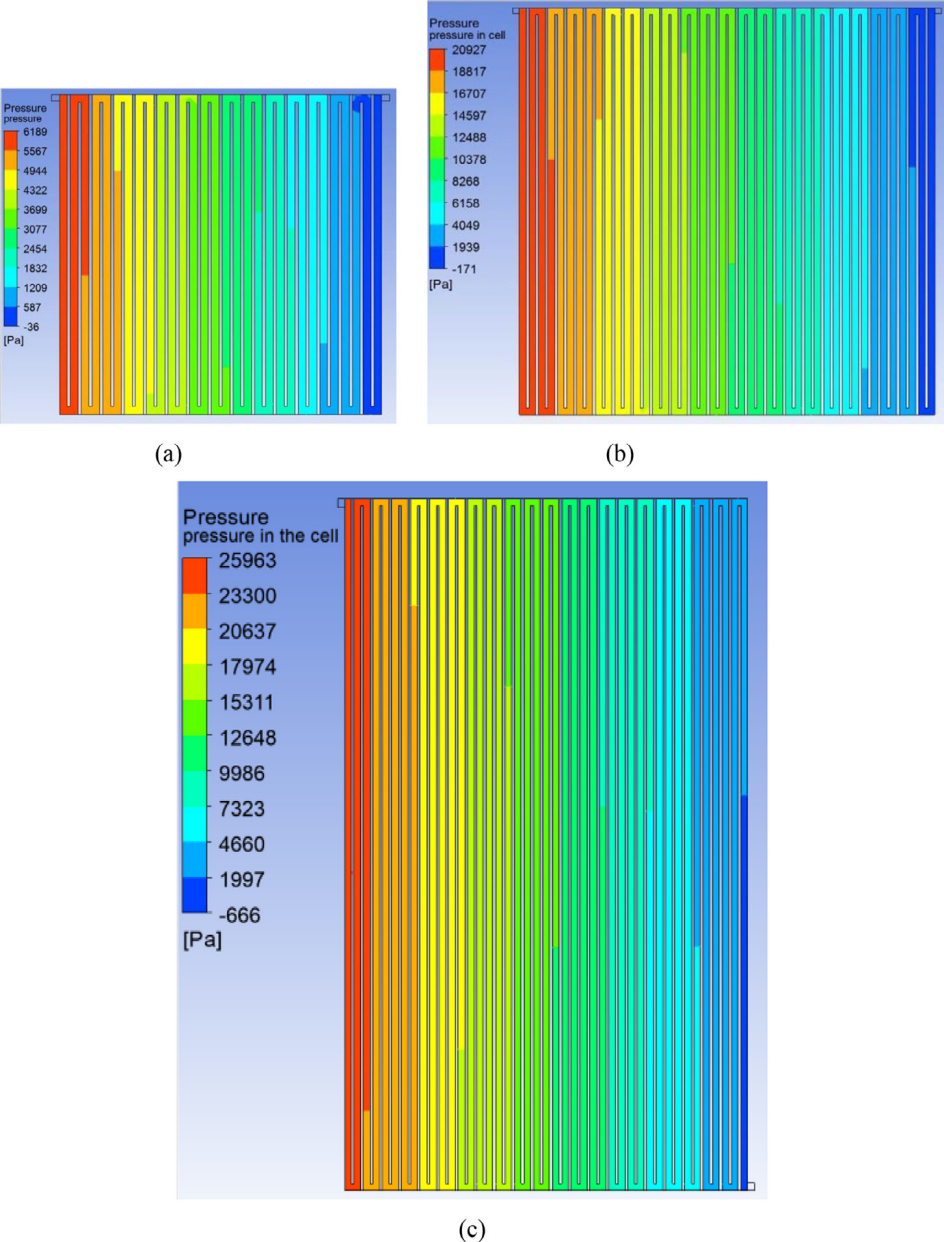
Predicted pressure contours for an inlet flow rate 0.30 ml/min.cm^2^ along a mid-plane of cell for the area (a) 416 cm^2^, (b) 918 cm^2^ and (c) 1495 cm^2^.

Fig. 6: CFD predicted pressure contours for an inlet flow rate 0.30 ml/min.cm^2^ along a mid-plane of cell serpentine flow field (a plane drawn at half depth of the flow-channel) for the areas 416 cm^2^, 918 cm^2^ and 1495 cm^2^.

**Fig. 7:**
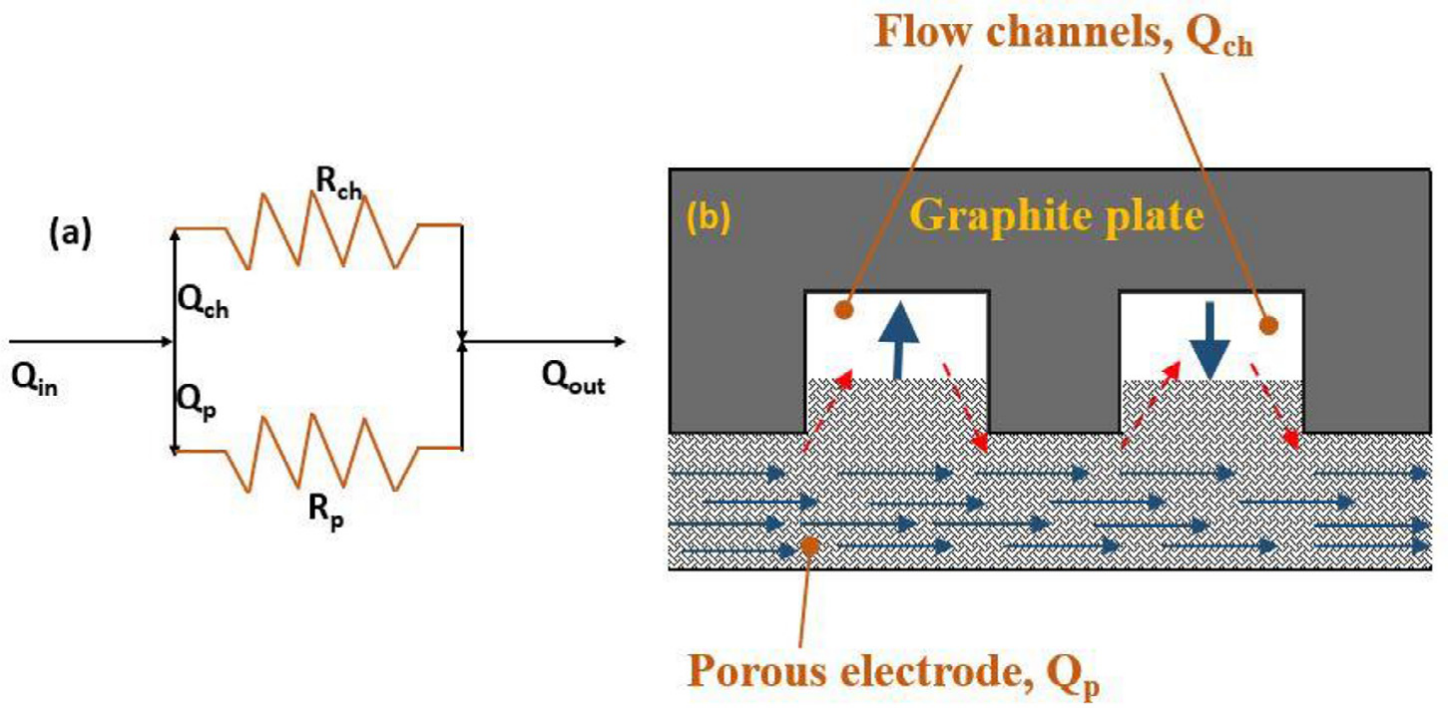
Schematic of parallel-path electrolyte circulation model showing (a) parallel split of inlet electrolyte flow in to two fluid streams flowing through flow-channel (Q_ch_) and porous zone (Q_p_) and (b) porous electrode attached to the serpentine flow field engraved on a graphite plate.

Fig. 7: Schematic showing the splitting of electrolyte flow between flow-channel and porous zone in a typical serpentine flow field.

Excel sheet 1: Raw and processed data_Research article 1_Serpentine scale up_400,900 and 1500 cm^2^ areas – it contains data of several life cycles conducted at various area specific flow rates (0.18 to 0.84 ml/min.cm^2^) and current densities (45 to 120 mA/cm^2^) for an electrode compression ratio of 35% and serpentine flow-channel dimensions (5 × 2 × 3) (channel width × rib width × channel depth) in mm. It also presents velocity profiles across the porous electrode and flow channels predicted by CFD simulations over the three cell areas for various flow rates.

Excel sheet 2: Raw data_Research article 2_Channel dimensions_400 and 900 cm^2^ areas – it contains raw data exported from BT-lab (biological) for the life cycles conducted for the operating condition 60 J SF6 (represents a current density (J) of 60 mA/cm^2^ with flow rate corresponding to stoichiometric factor (SF) of 6) for all the ten combinations of flow-channel dimensions of serpentine flow field.

Excel sheet 3: Processed data_Research article 2_Channel dimensions_400 and 900 cm^2^ areas – it contains measured pressure data at various flow rates, processed electrochemical parameters, power density data and average life cycles at the operating condition 60 J SF6 for all the ten combinations of flow-channel dimensions of serpentine flow field.

## 2 Experimental Design, Materials and Methods

### 2.1 Construction of cell

Several flow-channel dimensions of serpentine flow field as listed in Table 1 have been grooved on graphite plates of the dimension 30 cm × 30 cm × 1.5 cm in which the active area of the grooved channels was approximately 20 cm × 21 cm. Channel widths of 3, 4, 5 mm, depths of 3, 4 mm and rib widths of 2, 3 mm have been chosen for the study as listed in Table 1. Schematic of serpentine flow field is shown in Fig. 1. The graphite plates had given 5 cm as the overhead dimension on each side to accommodate compressible gasket (Fig. 1c) to arrest the leakage of electrolyte from active area. The studies of few flow-channel dimensions were extended to cell active area of 900 cm^2^ (30 cm × 30 cm). The optimal flow-channel configuration (5 × 2 × 3) (channel width × rib width × channel depth) has been studied systematically on three cell active areas namely 416 cm^2^ (20.8 cm × 20 cm), 918 cm^2^ (30.6 cm × 30 cm) and 1495 cm^2^ (29.9 cm × 30 cm) for various flow rates and current densities. Graphite felt (GFA 6EA from SGL Carbon) was placed on the active areas of the graphite plates (as both anode and cathode material), the membrane (Nafion 117) was sandwiched between the anode and cathode felt, copper plates of equal dimension as graphite plates were placed next to the them followed by end supporting plates which were insulated from the current carrying copper plates and the cell was tightened to the required level of electrode compression ratio (CR), which is defined in the Eq. (1), using suitable thickness of silicone gasket. An assembled single cell in operation and its components can be seen in Fig. 2.

(1) $$CR\;\left(\%\right)=\left(1-\;\frac{t_{c}}{t_{i}}\right)\times \;100\;$$

where *t_c_* is the thickness of felt after compression and *t_i_* is the initial thickness before compression.

### 2.2 Stoichiometric flow factor

The electrolyte circulation rate is defined in terms of stoichiometric flow factor (SF) which is based on the Faraday's laws of electrolysis. Molar stoichiometric requirement of electrolyte flow for an operating current is given in the Eq. (2).

(2) $$SF=\;\frac{i}{nFC}$$

where *i* = current (A), *n* = number of electrons participating in the reaction (=1 mole of e^−^/mole of solution for VRFB system), *F* = Faraday's constant (=96,486 A.s/mole e^−^), *C* = Concentration of electrolyte (mole/lit).

As the concentration of active reactant decreases with reaction, the theoretical stoichiometric flow requirement is defined by assuming 50% of the state of charge (SOC) of the active species. Therefore, the stoichiometric flow factor is defined as function of SOC as given in the Eq. (3).

(3) $$SF_{SOC}=\;\frac{i}{nFC}\times \frac{1}{SOC}$$

Therefore, a flow rate requirement based on Eq. (3) is named as stoichiometric flow factor of one (SF 1).

### 2.3 Measurement of pressure drop and pumping losses

Pressure drop in a cell was measured using an inverted U-tube manometer with air as the manometer fluid and U-tube manometer with mercury as the manometer fluid for the circulation of electrolyte and water, respectively. It was measured between the inlet of the cell and immediate outlet of the cell (between A and B in Fig. 3a). A T-junction was created near the inlet; one line of the T-junction enters the cell inlet and the other connected to one leg of manometer as shown in Fig. 3a. Similarly, using another T-junction at outlet, one line connected to the reservoir tank and the other to second leg of manometer. The difference in the levels of manometer legs is taken as the head loss and the corresponding pressure drop can be calculated by Eq. (4). The pressure drop between the entry and the exit sections, which was measured by piezometer (Fig. 3b), is subtracted from total cell pressure drop and only the pressure drop over the active area is reported in the present data.

(4) ΔP=(Δρ)g(ΔH)

where *ΔP* = pressure drop between inlet and outlet of the cell, Pa or kg/m.s^2^*Δρ* = difference in density between the manometer fluid and circulating fluid, kg/m^3^*g* = gravitation constant = 9.81 m/s^2^*ΔH* = manometer head, m.

The density of water, mercury, air and electrolyte solution (of concentration 1.61 M) are taken as 998.2, 13520, 1.225 and 1445 kg/m^3^ respectively.

If the pressure drop in a cell is known, then the power consumed by the pump operation can be calculated from the following equation

(5) $$P_{pump}=\;\frac{\left(\Delta P\right)\times Q}{\eta }\;$$

where ΔP = the cell pressure drop (Pa), *Q* = volumetric flow rate (m^3^/s), η = efficiency of the pump (taken to be 75%).

### 2.4 Measurement of electrochemical parameters

The electrolyte solution was prepared by dissolving 1.61 M vanadium oxysulfate (VOSO_4_. *x*H_2_O, *x* = 2 ∼ 2.3) in 5 M sulfuric acid. The raw electrolyte solution contained vanadium in VO^2+^ ionic state. The redox couple VO^2+^/VO^2^_+_and V^2+^/V^3+^ was obtained by two-step charging procedure as described in the reference number [3] with a pre-set voltage cut-off 1.8 V. Nitrogen gas was purged through the negative electrolyte. All experiments were conducted in an air-conditioned room maintained at 26 °C.

After completing dual-step charging process, the cell was programmed to run three life cycles for a given operating condition and the average of the three is considered in the processed data. Pre-set cut-off voltage limits 1.8 V for charging and 0.8 V for discharging were fixed for all the experiments. Electrochemical characteristics of a VRFB cell are described in terms of capacity, round-trip energy efficiency, coulombic efficiency, voltaic efficiency, net energy efficiency, energy density and power density. These terminologies are defined as follows

(6) Capacity=∫I(t)dt

(7) $$\text{Coulombic}\;\text{efficiency}\;\left(\text{CE}\right)=\int I_{d}\left(t\right)\;dt\;/\;\;\int I_{c}\;\left(t\right)\;dt$$

(8) $$\text{Round}-\text{trip}\;\text{energy}\;\text{efficiency}\;\left(\text{EE}\right)=\left\{\int I_{d}\left(t\right)\;E_{d}\left(t\right)\;dt\right\}\;/\;\left\{\int I_{c}\;\left(t\right)\;E_{c}\left(t\right)\;dt\right\}$$

(9) Voltaicefficiency(VE)=EE/CE

where *I* and *E* are the current and the voltage of the cell and the subscripts *c* and *d* denote charging and discharging phase of the cycle. From these, the system efficiency is calculated as follows:

(10) $$\text{Net}\;\text{energy}\;\text{consumed}\;\text{during}\;\text{charging}=W_{c}=\int \left\{I_{c}\left(t\right)\;E_{c}\left(t\right)\;+\;2\;P_{pump,c}\right\}\;dt$$

(11) $$\text{Net}\;\text{energy}\;\text{delivered}\;\text{during}\;\text{discharge}=W_{d}=\int \left\{I_{d}\left(t\right)\;E_{d}\left(t\right)-2\;P_{pump,d}\right\}\;dt$$

(12) $$\text{Net}\;\text{system}\;\text{efficiency}=W_{d}/W_{c}$$

Further, current density (*j*) and power density are defined based on unit nominal electrode area while the gravimetric or volumetric energy density of the electrolyte are defined based on the mass or the volume of the electrolyte on one side (anode/cathode) of the cell.

### 2.5 CFD simulations

The flow distribution including the magnitudes of pressure drop and velocity have been predicted through CFD simulations of flow through the graphite flow field attached to a compressed electrode. A 3-dimensional geometry of serpentine flow field as shown in Fig. 1 for the three cell areas was created using the design modeler of the CFD software ANSYS, version 18.1. Preliminary simulations were performed with various mesh sizes to establish grid independence. Mesh size of 0.25 mm was found to give grid-independent results and was therefore used for all simulations. A plane is drawn at mid-depths of flow-channel and porous electrode regions. Velocity and pressure contours have been obtained along these planes for an inlet flow rate 0.3 ml/min.cm^2^ and are shown in Figs. 4–6 for the cells of active area of 416, 918 and 1495 cm^2^. The cross-sectional area of inlet pipe is 12 mm^2^ in 416 cm^2^, 918 cm^2^ cells and 18 mm^2^ in the 1495 cm^2^ cell. For the operating flow rate mentioned above, the inlet velocities in the inlet pipe are 0.171 m/s, 0.381 m/s and 0.415 m/s for the small, medium and large cells respectively. Velocity profiles along the line drawn in width direction from inlet to outlet at the mid-depth of electrode and flow-channels are presented in the excel sheet 1.

### 2.6 Parallel-path electrolyte circulation model

CFD simulations of the type described in [1,2] for flow through the half-cell of a VRFB with a serpentine flow field attached to a compressed porous electrode show that the flow of electrolyte can be considered as having two parallel paths, one going through the serpentine channels grooved in the graphite plate and the other going through the porous electrode. The splitting of flow occurs in the first flow-channel and the two recombine near the common exit. In addition, across each pair of adjacent parallel channels, some under-the-rib convection occurs. This is illustrated as a schematic shown in the Fig. 7. As also shown in Fig. 1 of research article [2], one can see large velocity flow that occurs in the axial direction in the flow-channels and the much smaller velocity flow in the electrode that occurs in the transverse direction. Within the electrode, there is a strong undercurrent that flows all the way from the first serpentine channel to the last one. In addition, near the interface of flow-channel and electrode, local circulation into and from the channel is also present. However, this fluctuation in the flow rate through the electrode arising from local circulation is neglected in the model. Detailed analysis of CFD results for various configurations shows the variable component of the substrate flow is rather small (typically less than 10%) of the total substrate flow for typical serpentine flow fields. In view of this, only the steady component of the substrate flow, which is the result of the overall pressure drop across the cell, is considered. Detailed mathematical description of the model and its validation with experimental results is presented in research article [2]. The model can be used to calculate the cell pressure drop for a given circulation rate and vice versa.

## Declaration of Competing Interest

The authors declare that they have no known competing financial interests or personal relationships which have, or could be perceived to have, influenced the work reported in this article.
